# Physical Activity and Physical Function One Year After Hospital Discharge for COVID-19

**DOI:** 10.3390/jcm14176206

**Published:** 2025-09-02

**Authors:** Eva Arents, Fien Hermans, Lies Glorie, Bihiyga Salhi, Cedric Bosteels, Eric Derom, Wim Janssens, Eva Van Braeckel, Natalie Lorent, Yannick Vande Weygaerde, Thierry Troosters, Heleen Demeyer

**Affiliations:** 1 Department of Rehabilitation Sciences, Ghent University, 9000 Ghent, Belgium; eva.arents@ugent.be (E.A.); fien.hermans@ugent.be (F.H.); 2 Department of Rehabilitation Sciences, KU Leuven, 3000 Leuven, Belgium; lies.glorie@uzleuven.be; 3 Department of Respiratory Medicine, Ghent University Hospital, 9000 Ghent, Belgium; bihiyga.salhi@ugent.be (B.S.); cedric.bosteels@ugent.be (C.B.); eric.derom@ugent.be (E.D.); eva.vanbraeckel@ugent.be (E.V.B.); yannick.vandeweygaerde@uzgent.be (Y.V.W.); 4 Department of Internal Medicine and Paediatrics, Ghent University, 9000 Ghent, Belgium; 5 Department of Respiratory Diseases, University Hospital Leuven, 3000 Leuven, Belgium; wim.janssens@uzleuven.be (W.J.); natalie.lorent@uzleuven.be (N.L.); 6 Laboratory of Respiratory Diseases and Thoracic Surgery (BREATHE), Department of Chronic Diseases and Metabolism, KU Leuven, 3000 Leuven, Belgium

**Keywords:** activities of daily living, COVID-19, health status, hospitalization, intensive care units

## Abstract

**Background:** Immediately after discharge from hospital, COVID-19 patients have poor physical function and impaired performance in activities of daily living. Persisting symptoms and cognitive impairments have been reported, but the long-term impact on objectively measured physical activity (PA) in patients hospitalized for COVID-19 is not clear. **Methods:** A prospective cohort study was conducted to compare objectively measured PA and physical function 12 months post discharge in patients who were hospitalized for COVID-19 with age- and sex-matched healthy controls and to elucidate the impact of ICU admission on these outcomes. PA was objectively assessed using accelerometry in patients, healthy controls, and in a subset of partners of patients. Additionally, lung function, physical function (six-minute walk distance (6 MWD) and isometric quadriceps and handgrip force), symptom experience, and health-related quality of life (HRQoL) were evaluated in patients with and without ICU admission. **Results:** Included in the study were 101 patients (60 ± 10 years, 69% male), 36 healthy controls (60 ± 9 years, 58% male), and 14 partners (55 ± 8 years, 21% male). Daily step count and movement intensity (MI) during walking in patients were significantly lower compared with healthy controls (6726 ± 328 vs. 8155 ± 555 n.day^−1^, *p* = 0.03 and 1.99 ± 0.04 vs. 2.21 ± 0.07 min/s^2^.day^−1^, *p* = 0.005). PA levels of patients and their partners were comparable. Physical function, symptom experience, HRQoL, and PA levels were comparable in patients with and without ICU admission (*p* > 0.05). Daily step count was weakly positively associated with 6 MWD (r = 0.30). **Conclusions:** One year post discharge, patients had lower PA levels than healthy controls. ICU admission did not affect physical function, symptoms, HRQoL or activity levels.

## 1. Introduction

The coronavirus disease 2019 (COVID-19) pandemic has had an enormous global impact with over 770 million confirmed cases of SARS-CoV-2 infections and over 7 million deaths as of 20 March 2025 [1]. Despite a relatively high mortality rate in the acute phase, most patients recovered, but the long-term impact of a SARS-CoV-2 infection is not well understood. Even though COVID-19 mainly targets the respiratory system, the possibility of other organs being affected has been widely observed [2].

Patients hospitalized for COVID-19 have been shown to have poor physical function and impaired performance in activities of daily living (ADLs) immediately after discharge [3]. Subsequently, a significant proportion of patients experience long lasting symptoms and impairments. Several cohort studies reported sequalae, including fatigue, anxiety, muscle weakness, sleep disturbance, hair loss, decreased appetite, smell disorder, palpitations, headache, cognitive impairment and dyspnea up to 3 to 12 months after discharge for COVID-19 [4,5,6,7]. Persistent changes on pulmonary and physical function, as well as abnormalities on chest computed tomography (CT) have been noted, mostly in patients who were critically ill during hospital stay [4]. When patients experience long lasting symptoms and impairments, this condition is called long COVID or post-acute COVID-19 syndrome (PACS) and is defined as ‘a set of signs and symptoms that emerge during or after an infection consistent with COVID-19, persist for more than 12 weeks, and are not explained by an alternative diagnosis [8]. Hence, PACS can be considered a multisystem syndrome that may require full multidisciplinary rehabilitation to enable adaptation. Research demonstrated exercise-based rehabilitation to be an effective and safe strategy to reduce symptoms and improve exercise capacity and QoL [9].

However, few studies have objectively evaluated the extent to which such impairments persist, particularly in terms of PA levels. Previous studies reported low self-reported PA up to 18 months in patients post COVID-19 [10,11,12,13]. This was confirmed by objective measurements of PA, which ranged from 7 min.day^−1^ to 60 min.day^−1^ of moderate-to-vigorous PA (MVPA) or 4513 steps/day in patients 4–18 months post COVID-19 [14,15,16,17,18]. However, only two small studies compared objectively measured PA levels after COVID-19 with healthy controls [19,20].

During the overwhelming COVID-19 pandemic, many patients were admitted to the ICU. Patients who survive critical illness have been shown to be at risk for persistent physical impairments associated with Post Intensive Care Syndrome (PICS) [21]. However, the further impact of ICU admission has not been elucidated regarding PA and the association with physical function after COVID-19.

The primary aims of the present study were to characterize the objectively measured PA in a cohort of previously hospitalized patients with moderate (no need for ICU admission) to severe (need for ICU admission) COVID-19 in Belgium at 12 months post discharge and to compare these PA outcomes with age- and sex-matched healthy controls and to additionally compare PA outcomes in a subset of patients with these of their own partners. Secondly, the impact of ICU admission and associations between physical function and PA were investigated in the patient cohort. We hypothesized that 12 months after discharge, patients exhibited lower PA levels compared with healthy controls, especially in those following ICU admission.

## 2. Methods

### 2.1. Study Design and Patient Population

The present prospective cohort study with an age- and sex-matched comparison group is a sub-study of a large cross-sectional observational multicenter cohort study, which was approved by the institutional ethics committee of Ghent University Hospital (BC-09986, BC-10247) and the institutional ethics committee of University Hospital Leuven (S64081; S65411). Results on part of this cohort were published previously [22]. Adult patients admitted to either University Hospital Leuven (UZL) or Ghent University Hospital (UZG) with COVID-19 (confirmed by SARS-CoV-2 RT-PCR and/or CT of lungs) between March 2020 and September 2021 were included in the large cohort study. Some of the patients from this cohort who were scheduled for a 12-month outpatient follow-up consultation were invited for the present sub-study and seen in the outpatient clinic 12 months after discharge between April 2021 and July 2022.

Simultaneously with recruitment of the last patients, recruitment of a cohort of healthy age- and sex-matched controls was initiated in July 2021 and finalized in October 2022. Additionally, all partners of the included patients between April 2021 and July 2022 were also invited to participate. The healthy control group and the partners of patients, were 18 years of age or older, had never been diagnosed with COVID-19 requiring hospitalization, were asymptomatic at inclusion (in the case of individuals who did experience COVID-19 but did not require hospitalization)—and had no comorbidities affecting physical activity in daily life. The healthy control group was recruited from the authors’ social network.

The present study consisted of a single clinical visit and a follow-up period of 7 days as part of the standard outpatient clinical visit at 12 months post discharge. Procedures of and criteria for data collection of patients and healthy controls were aligned prior to implementation in both centers. Prior to data collection, written informed consent was obtained from patients, healthy controls, and partners willing to participate.

The paper was reported according to the Strengthening the Reporting of Observational studies in Epidemiology (STROBE) guideline.

### 2.2. Physical Activity Assessment

During the 7-day period following the clinical visit at 12 months, PA data were collected in both groups by accelerometry. The DynaPort MoveMonitor (DAM, McRoberts, The Hague, The Netherlands) is a small (106.6 × 58 × 11.5 mm), light-weight CE-marked tri-axial accelerometer with a sampling frequency of 100 Hz, validated to measure PA objectively [23,24]. All subjects were asked to wear the monitor during waking hours and only remove the device during water-based activities. The measurement was valid when the DAM registered data for at least two days, eight hours per day [23,24]. The following outcomes were retrieved: daily step count, daily walking time, and daily movement intensity during walking (MI). MI is expressed in m/s^2^ and reflects the average acceleration during ambulation episodes and is considered a proxy for walking vigor or cadence. This metric has previously been assessed in patients with Chronic Obstructive Pulmonary Disease (COPD) using the DAM [25]. Outcomes were presented as absolute values. Additionally, the proportion of patients and healthy controls meeting different levels of PA based on daily step count was shown (<7500 daily step count: ‘low active to sedentary’; ≥7500 daily step count: ‘somewhat active to active’) [26].

### 2.3. Clinical Outcomes and Subject Characterization

At the clinical visit, the following assessments were performed in both groups: (1) Sociodemographic data (age, sex, height, and weight); (2) smoking history; and (3) work status. In patients, the following additional measurements were performed: (1) Lung function, including spirometry and diffusing capacity for carbon monoxide (DLco), in accordance with ERS/ATS recommendations [27] and with impairment defined as <80% predicted values; (2) functional exercise capacity based on a six-minute walk test in accordance with ERS/ATS guidelines (normal values for the six-minute walk distance (6 MWD) were used, and impairment was defined as ≤70% of predicted values); (3) peripheral muscle strength by maximal isometric quadriceps muscle force using a fixed strain gauge dynamometer and handgrip muscle force using a Jamar Hydraulic Hand Dynamometer (JA Preston Corporation, Jackson, MI, USA) according to a standardized protocol [28,29] (the best of 3 attempts was reported relative to body weight and normal values, and impairment was defined as ≤70% of predicted values); (4) the Modified Medical Research Council scale (mMRC) questioning dyspnea; (5) the Short Form Health Survey (SF-36) questioning perceived health and HRQoL (the physical component summary and mental component summary were calculated and reported as norm-based scores—a mean score of 50 is considered normal and a difference of 10 is clinically relevant—based on USA normative scores [30]); (6) the Hospital Anxiety and Depression Scale (HADS) [31]; and (7) a rehabilitation trajectory. More information can be found in the previous publication [22].

### 2.4. Statistical Analysis

All statistical analyses were performed using the IBM SPSS statistics version 29 (IBM Corp., Armonk, NY, USA). Data are presented as mean ± standard deviation, median (Q1; Q3), or percentages, as appropriate after testing for normality using the Shapiro–Wilk test and inspecting QQ-plots. Statistical significance was set at *p* < 0.05 for all analyses.

PA outcomes between patients and healthy controls were compared using ANCOVA. Because of the known seasonal effect, analyses were adjusted for duration of daylight as a proxy for season [24]. To assess the robustness of group comparisons, bootstrapped ANCOVA models (1000 resamples, BCa 95% CI), adjusted for daylight hours, were performed for PA outcomes. PA outcomes between patients and their partners were compared using the independent Samples T-test, and their associations were analyzed via Pearson correlation with 95% confidence intervals, based on 1000 bootstrap samples. The independent Samples T-test (normally distributed) or the Mann–Whitney U Test (not normally distributed) were used for other continuous data. Categorical data were compared with Chi-square tests. To compare COVID-19 patients with and without an ICU-stay, ANCOVA was adjusted for duration of daylight (PA), and the independent Samples T-test or the Mann–Whitney U Test were used according to the data distribution. For ANCOVA, estimated marginal means (EMMs) and standard errors (SEs) are reported. For all primary and key secondary continuous outcomes, the absolute mean difference between groups and its 95% confidence interval were calculated to aid the interpretation of clinical relevance.

Associations between physical function and PA outcomes of patients were evaluated via Pearson correlation with 95% confidence intervals, based on 1000 bootstrap samples. Correlation strength was interpreted following Mukaka’s guideline [32]: r < 0.30 as negligible, 0.30–0.50 as weak, 0.50–0.70 as moderate, 0.70–0.90 as strong, and >0.90 as very strong correlation. Correlation strength was interpreted based on the absolute value of r, treating positive and negative correlations equivalently.

## 3. Results

**Figure 1:**
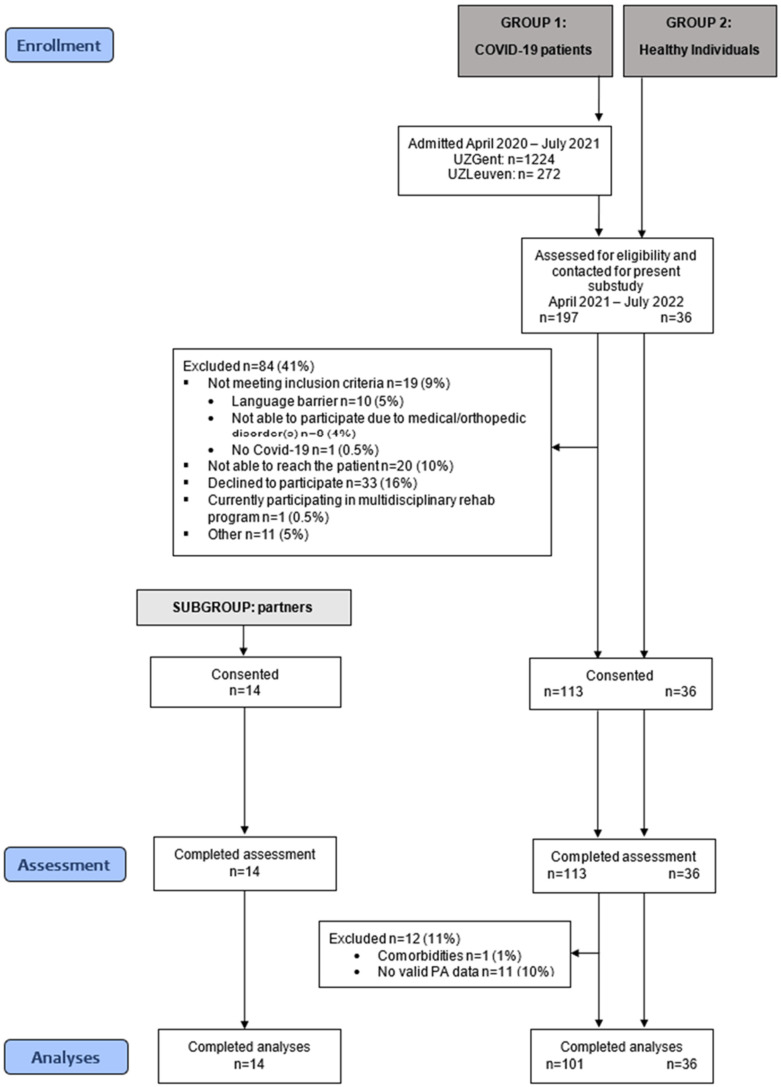
Flow diagram of recruitment.

197 patients who had been included in the large cohort study between April 2021 and July 2022 were screened and contacted (Figure 1) to participate in the current sub-study. The consent rate was 67%. A total of 113 patients completed the assessments. One patient was excluded from the analyses due to comorbidities hampering performance during the physical test battery. In addition, 36 age- and sex-matched healthy controls were included. Partners from all included patients were also asked to participate, of which 14 partners consented and were included. From this final grouping, valid PA data were obtained in 101 patients, 36 healthy controls and a subset of 14 patient–partner dyads.

### 3.1. Baseline Characteristics

Baseline characteristics of the 101 included patients and 36 age- and sex-matched healthy controls are shown in Table 1. Patients had a significantly higher BMI (28.7 (26.4; 32.8) kg/m^2^ vs. 24.8 (23.6; 29.0) kg/m^2^, *p* < 0.001) compared with healthy controls, and significantly fewer patients were active smokers (3% vs. 14%, *p* = 0.02). Of the patients and the healthy controls, 53% and 75%, respectively, were included during winter/autumn. A longer duration of daylight (proxy for season) during the PA assessment of patients was observed as compared with healthy controls (715 ± 176 vs. 636 ± 202 min.day^−1^, *p* = 0.03).

There were 14 patient–partner dyads included in the study, and their baseline characteristics are presented in Table 2. During winter/autumn, 71% of the dyads were assessed; during spring/summer, 29% of the dyads were assessed.

### 3.2. Physical Activity

#### 3.2.1. PA of Patients and Healthy Controls

**Figure 2:**
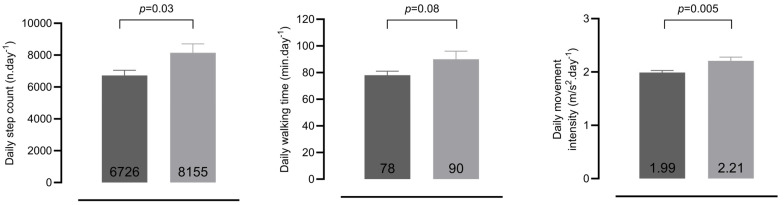
Bar charts of physical activity of COVID-19 patients (*n* = 101) and healthy controls (*n* = 36). Data are presented as estimated marginal means, and the error bars represent the standard error. The dark gray bars represent the patient group, and the light gray bars represent the healthy control group. ANCOVA analyses, adjusted for duration of daylight (proxy for season), were performed.

Daily step count and daily MI were significantly lower in patients compared with age- and sex-matched healthy controls (6726 ± 328 vs. 8155 ± 555 n.day^−1^, *p* = 0.03 and 1.99 ± 0.04 vs. 2.21 ± 0.07 min/s^2^.day^−1^, *p* = 0.005, respectively) (Figure 2). The absolute mean difference for step count was −1429 n.day^−1^ (95% CI: −2679 to −179) and −0.22 m/s^2^.day^−1^ (95% CI: −0.37 to −0.07) for MI. There was a trend towards shorter daily walking time in patients compared with healthy controls (78 ± 3 vs. 90 ± 6 min.day^−1^, *p* = 0.08), with a mean difference of −12 min.day^−1^ (95% CI: −25 to 1). These differences remained consistent throughout the bootstrapped analyses (Supplementary Table S1).

#### 3.2.2. PA of Patient–Partner Dyads (*n* = 14)

Daily step count, daily walking time, and daily MI were comparable between patients and their partners (6670 ± 3999 vs. 5801 ± 2260 n.day^−1^, *p* = 0.49; 76 ± 40 vs. 67 ± 24 min.day^−1^, *p* = 0.48; 2.04 ± 0.34 vs. 2.14 ± 0.49 m/s^2^.day^−1^, *p* = 0.55, respectively). The absolute mean differences were 896 n.day^−1^ (95% CI: −1796 to 3534), 9 min.day^−1^ (95% CI: −20 to 39), and −0.10 m/s^2^.day^−1^ (95% CI: −0.45 to 0.25), respectively. A significant moderate correlation was found between daily step count and MI of patients and their partners (r = 0.64, *p* = 0.01, 95% CI: 0.16 to 0.87 and r = 0.57, *p* = 0.03, 95% CI: 0.05 to 0.84, respectively).

### 3.3. The Long-Term Impact of an ICU-Stay

The clinical characteristics, physical functioning, PA, and symptom experience of patients with an ICU-stay (*n* = 45) and patients without an ICU-stay (*n* = 56) are presented in Table 3. Patients that were admitted to the ICU ward (10.0 (5.0; 19.5) days in the ICU) had a higher BMI (30.3 (27.2; 35.1) vs. 28.1 (25.3; 30.9) kg/m^2^, *p* = 0.024) and a longer length of hospital stay (LOS) (*p* < 0.001). More patients with an ICU-stay were male (*p* < 0.001), and 61% of patients admitted to the ICU required invasive mechanical ventilation (IMV). Patients admitted to the ICU tended to be older (*p* = 0.07), and significantly more patients in the ICU group presented with a diffusion capacity below 80% of the predicted normal value (42% vs. 22%, *p* = 0.03) after 12 months.

Differences between groups in terms of daily step count and daily walking time were not observed (*p* = 0.90, *p* = 0.76), and daily MI during walking tended to be lower in the group admitted to the ICU (*p* = 0.09). Functional exercise capacity, quadriceps force, handgrip force, and symptom experience were comparable between groups. Overall, approximately one-third of patients presented with impaired quadriceps force, whereas exercise capacity and HRQoL were normal at the group level.

### 3.4. Associations Between Physical Function and Physical Activity in COVID-19 Patients

**Figure 3:**
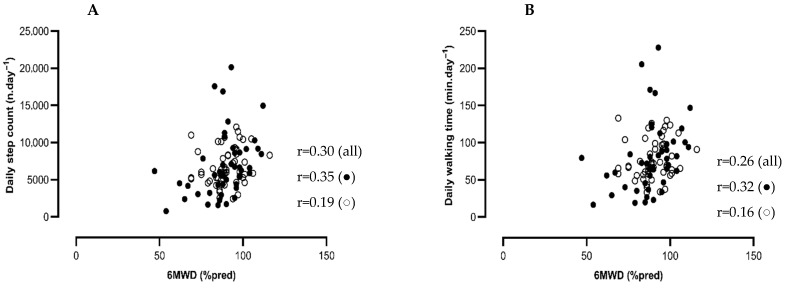
Scatter plots of associations between daily step count and 6 MWD (% pred) (**A**) and associations between daily walking time and 6 MWD (% pred) (**B**). Patients with an ICU-stay are depicted in the closed dots, and patients without an ICU-stay are depicted in the open dots. Pearson correlations are performed.

Average daily step count was weakly but significantly associated with 6 MWD (% pred) (r = 0.30, *p* = 0.002, 95% CI: 0.11 to 0.47) (Figure 3) but not with QF (% pred) or HGF (% pred) (r = 0.05, *p* = 0.62, 95% CI: 0.16 to 0.26 and r = 0.01, *p* = 0.95, 95% CI: −0.19 to 0.20, respectively). Similar associations were found for walking time (r = 0.26, *p* < 0.01, 95% CI: 0.07 to 0.44; r = 0.06, *p* = 0.58, 95% CI: −0.15 to 0.26; and r = 0.03, *p* = 0.77, 95% CI: −0.17 to 0.23 for 6 MWD (% pred) (Figure 3), QF (% pred), and HGF (% pred), respectively). Average daily MI was not associated with 6 MWD (% pred), QF (% pred), or HGF (% pred).

## 4. Discussion

To the best of our knowledge, this is the first relatively large study comparing objectively measured PA between patients at one year after a hospital admission for moderate to severe COVID-19 and an age- and sex-matched healthy control group. Our findings showed that daily step count and movement intensity of COVID-19 patients were significantly lower compared with healthy controls, and a trend towards shorter daily walking time was observed. Partners of patients had a comparable low physical activity (PA) level.

In a post SARS-CoV-2 infection study, Delbressine et al. [10] showed a significant decrease in self-reported measured walking time at 3 months post infection as compared with pre-infection levels and only a partial recovery at 6 months post infection, with self-reported measured walking time of 90 (Q1; Q3, 30; 150) min.week^−1^. Objective data from our study showed higher levels of PA (70 (Q1; Q3, 57; 98) min.day^−1^) at 12 months post discharge. Since the PA levels reported by Delbressine et al. are based on subjective measurements, PA may have been underestimated, which may perfectly explain the discrepancy with our objectively measured PA outcomes. Unfortunately, the design of our study does not allow for a comparison with pre-COVID-19 PA levels, which limits our ability to establish whether the reduced PA observed was a consequence of the infection and hospitalization or, rather, a reflection of pre-existing behavior. Interestingly, self-reported pre-infection PA has been shown to be the strongest risk factor for hospital admission, ICU admission, and death as result of a SARS-CoV-2 infection [33].

In our patient sample, we observed an average step count of 6726 n.day^−1^12 months after discharge. This was higher than the 5306 steps reported by Diciolla et al. [18] at the same time point. The discrepancy could be the result of a difference in the type of activity monitor used. In contrast to our study design, these authors did not include a comparison with a healthy control group, which makes the interpretation of results more difficult. The lower PA could also be explained by the patient cohort. Whereas general patient characteristics (age, BMI, and gender) were very similar, the proportion of patients with functional impairment (<70% pred 6 MWD and QF) was higher in the paper of Diciolla et al. [18]. Previously, smaller studies have assessed objectively measured PA in patients versus healthy controls. Tryfonos et al. [19] reported significantly lower MVPA (34.8 vs. 61.3 min.day^−1^) in 31 patients at 9 months post hospitalization compared with 31 healthy controls. Freire et al. [20] reported that MVPA tended to be lower in the 20 patients at 1.5 months post infection compared with the 18 healthy controls (14.8 vs. 23.7 min.day^−1^) in their study. Similarly, the PA levels of the patients were lower than those of the healthy controls, with significant differences observed in daily step count and movement intensity during walking (−1429 n.day^−1^, *p* = 0.03; and −0.22 min/s^2^.day^−1^, *p* = 0.005, respectively). Notably, the observed difference in step count approaches the Minimally Clinically Important Difference (MCID) of 1000 steps.day^−1^ reported in patients with COPD [24], suggesting that the reduced activity levels in our post-COVID-19 cohort may be clinically meaningful. The PA levels in our healthy control group were comparable to those reported in previous studies using the same activity monitor [34], further supporting the validity of these findings. However, among the partners of the included patients, lower PA levels were observed compared with these previous samples, resulting in values similar to those of their partners with post-COVID-19. A moderate positive correlation in PA outcomes between patients and their partners was found, which may explain these lower values. This could suggest that living with a post-COVID-19 patient might impact the partner’s PA behavior due to shared lifestyle patterns or mutual activity habits [35]. While the inclusion of patient–partner dyads is a novel and potentially insightful approach, this subgroup was relatively small (*n* = 14). Therefore, these findings should be interpreted with caution and considered exploratory in nature. A longer duration of daylight hours during PA assessment was observed in patients as compared with healthy controls, leading to potentially higher PA levels in patients and smaller differences compared with controls. To address this, all PA analyses were adjusted for duration of daylight as a proxy for season.

Movement intensity adds value beyond step count or duration, as it captures the intensity of ambulatory movements [25]. Our data show a significantly lower movement intensity during walking in patients, suggesting a slower or less vigorous walking pattern. Whereas we cannot compare this information with any previous research, this finding is similar to the reduced walking speed previously reported in chronic respiratory populations such as COPD [25].

In other coronavirus-induced syndromes such as SARS and MERS, impaired diffusion capacity is a known long-term sequela. Recovery appears, but impaired diffusion capacity has been shown to last for months, even years, after discharge [36]. Huang et al. reported a diffusion capacity <80% predicted in 23% of patients 12 months post COVID-19 [4]. In our cohort, this was observed in 31%, with a significantly higher prevalence among ICU-treated patients.

Most patients showed good overall physical recovery, although about one-third had residual quadriceps weakness one year post discharge. This pattern is broadly in line with findings by Huang et al. [4], who similarly reported a limited number of severe physical impairments. We hypothesized that an ICU-stay and a longer length of hospital stay would lead to ‘ICU-acquired’ weakness in these patients, mainly affecting the lower limb muscles [37]. Surprisingly, large differences in physical recovery between patients with and without an ICU-stay at 12 months were not observed, in contrast with our initial hypothesis. To our surprise, one-third of patients had an impaired quadriceps force irrespective of an ICU-stay, which contrasts with data obtained in the same cohort 3 months post discharge. In that cohort, patients hospitalized with severe infection (98% with an ICU-stay) displayed more evidence of impaired quadriceps force compared with patients with moderate infection (7.8% with an ICU-stay) (59 vs. 69 % pred) [22]. Quadriceps force apparently recovered in the subsequent months. Several factors may explain why ICU admission was not associated with worse long-term outcomes. First, ICU admission criteria during the COVID-19 pandemic may have varied across regions and were possibly influenced by healthcare system pressures. In our centers, ICU admission thresholds were relatively low, potentially resulting in a less severely ill ICU cohort. In our sample, 45% of patients had an ICU-stay compared with only 4% in the cohort study by Huang et al. [4], and the median ICU length of stay in our cohort was also shorter. Second, survival bias may have influenced the results. Patients with more favorable recovery potential were more likely to survive ICU care and participate in follow-up assessments. Third, early rehabilitation interventions during or shortly after ICU discharge may have mitigated some of the expected long-term deficits. However, we cannot investigate this due to a lack of information on rehabilitation trajectories. Together, these factors could have attenuated differences in physical recovery between ICU and non-ICU patients.

In our patient cohort, daily step count was weakly positively associated with functional exercise capacity (6 MWD, % pred). This association was weaker than the associations reported in chronic respiratory diseases such as COPD (r = 0.5) [38]. This could be explained by the small spectrum of functional exercise capacity (6 MWD) hovering with the ceiling effect of the test (89 ± 12% pred) in the present cohort. In COPD, an improvement of 6 MWD after rehabilitation does not immediately lead to an enhancement of daily PA [39,40]. Similarly, exercise training after COVID-19 has no effect on PA levels [14]. In our patient cohort, 42% did follow some sort of rehabilitation.

## 5. Strength and Limitations

The present study has several strengths. This is the first relatively large study comparing objectively measured PA levels in patients hospitalized for moderate to severe COVID-19 12 months after hospital discharge with healthy controls. Patients and healthy controls were assessed within the same time window (2021–2022), which eliminates the impact that the varying pandemic-related restrictions possibly could have had on PA levels. Within this time period, patients admitted to hospital in both the first wave (March 2020–January 2021) and the second wave (March 2021–June 2021) of the pandemic were assessed.

Some limitations should be considered. As previously mentioned, patients and healthy individuals were age and sex matched, but a longer duration of daylight during the PA assessment was observed in patients compared with healthy controls. To account for this, all analyses were properly corrected for duration of daylight as a proxy for season [24]. Secondly, our findings cannot be compared with any pre-COVID-19 measurements since no objective or subjective data on the pre-infection state of patients were collected. Therefore, it cannot be excluded that the observed difference in daily step count and movement intensity would already have been present before the hospitalization. It might have been that the PA levels of COVID-19 patients and their partners were lower than the general population. Thirdly, patients scheduled for the standard one-year follow-up measurement in the hospital were invited to participate in this present study. Patients were not selected based on having post-COVID-19 symptoms or diagnosed PACS, which can explain why limited functional and symptom impairments were found in our patient sample. Because only a small amount of data was collected on symptoms (e.g., fatigue), it is not possible to distinguish whether the impairments are a result of PACS of PICS. Fourth, the healthy control group was recruited through the investigators’ social network, which may have introduced selection bias and limited the generalizability of comparisons. Nonetheless, the PA levels in our healthy control group aligned closely with those reported in previous studies applying the same activity monitor [34]. Additionally, the number of healthy controls was relatively small (*n* = 36), which may have reduced the statistical power needed to detect smaller between-group differences. To address this, we performed bootstrap ANCOVA analyses for the primary PA outcomes, adjusting for daylight hours (Supplementary Table S1). These analyses confirmed statistically significant differences for step count and movement intensity, thereby supporting the robustness of our findings despite the sample size limitation. Lastly, while we were able to document whether participants received any rehabilitation after hospitalization, we lacked detailed information on the type, timing, and intensity of these interventions. This limits our ability to assess the influence of rehabilitation on long-term outcomes. Previous research has already shown this possible influence during hospitalization [41] as well as post hospitalization [42]. International analyses emphasized the role of early, multidisciplinary rehabilitation—as demonstrated in Italian cohorts—in improving muscle strength, reducing dyspnea, and promoting independence during acute care [43]. These observations suggest that the lack of a standardized rehabilitation pathway in our cohort may have contributed to the persistent impairments observed.

## 6. Conclusions

Daily step count and daily movement intensity during walking in COVID-19 patients were significantly lower compared with age- and sex-matched healthy controls. Overall, an ICU-stay did not result in a different long-term recovery trajectory in patients with COVID-19 in this cohort.

## Figures and Tables

**Table 1:** Baseline characteristics of patients and healthy controls.

Characteristics	Patients (*n* = 101)	Healthy Controls (*n* = 36)	*p*-Value
Sex (*n* (% male))	70 (69)	29 (58)	0.17
Age (years)	60 ± 10	60 ± 9	0.71
BMI (kg/m^2^)	28.7 (26.4; 32.8)	24.8 (23.6; 29.0)	<0.001
Active smoker (*n* (%))	3 (3)	5 (14)	0.02
Work status (*n* (%))			0.75
- active	59 (58)	20 (59)	
- non-active	10 (10)	2 (6)	
- retired	32 (32)	12 (35)	
Season of inclusion (*n* (%))			0.10
- winter	19 (19)	9 (25)	
- spring	35 (35)	5 (14)	
- summer	13 (13)	4 (11)	
- autumn	34 (34)	18 (50)	

Data are presented as mean ± SD or median (Q1; Q3) unless otherwise indicated. BMI: Body Mass Index.

**Table 2:** Baseline characteristics of patient–partner dyads (*n* = 14).

Characteristics	Patients (*n* = 14)	Partner Healthy Controls (*n* = 14)	*p*-Value
Sex (*n* (% male))	11 (79)	3 (21)	0.002
Age (years)	58 ± 9	55 ± 8	0.36
BMI (kg/m^2^)	27.7 (24.7; 30.5)	28.2 (22.5; 32.0)	0.86
Active smoker (*n* (%))	0 (0)	2 (14)	0.14
Work status (*n* (%))			0.63
- active	8 (57)	9 (64)	
- non-active	2 (14)	3 (21)	
- retired	4 (28)	2 (14)	

Data are presented as mean ± SD or median (Q1; Q3) unless otherwise indicated. BMI: Body Mass Index.

**Table 3:** Clinical characteristics, physical activity, physical function, and symptom experience of COVID-19 patients with and without an ICU-stay at 12 months post discharge.

	All Patients	ICU	Non-ICU	Absolute Mean Difference (ICU—Non-ICU)	95% CI	*p*-Value *
N (*n* (%))	101 (100)	45 (45)	56 (55)			
Male (*n* (%))	70 (71)	39 (87)	31 (55)			<0.001
**Clinical characteristics**						
Age (years)	60 ± 10	62 ± 8	59 ± 10			0.07
BMI (kg/m^2^)	28.6 (26.4; 32.8)	30.3 (27.2; 35.1)	28.1 (25.3; 30.9)			0.02
FEV_1_ < 80%pred (*n* (%))	8 (8)	5 (11)	3 (5)			0.29
DL_CO_ < 80%pred (*n* (%))	31 (31)	19 (42)	12 (22)			0.03
LOS (days)	11.0 (8.0; 21.5)	23.5 (12.0; 39.0)	8.5 (6.0; 10.3)			<0.001
NIVM (*n* (%))	55 (56)	30 (68.2)	25 (46)			0.02
NIVM (days)	7 (5; 11)	9 (5; 15)	7 (5; 10)			0.14
IVM (*n* (%))	26 (27)	26 (61)	0			<0.001
Rehabilitation (*n* (%))	42 (42)	22 (49)	20 (36)			0.13
**Physical activity**						
Daily step count (n.day^−1^)	6800 ± 337	6759 ± 451	6841 ± 503	−82	−268 to 104	0.90
<7500 daily step count (*n*, %)	68 (67)	30 (67)	38 (68)			0.90
Daily walking time (min.day^−1^)	78 ± 4	80 ± 5	77 ± 5	−3.6	−18.5 to 11.3	0.76
Daily MI during walking (m/s^2^.day^−1^)	1.99 ± 0.04	1.92 ± 0.05	2.04 ± 0.47	−0.12	−0.40 to 0.17	0.09
**Physical function**						
6 MWD (m)	591 ± 108	582 ± 119	599 ± 98	−17	−61 to 27	0.44
6 MWD ≤ 70%pred (*n*, %)	8 (8)	5 (11)	3 (6)			0.30
QF (Nm/kg)	1.70 ± 0.50	1.66 ± 0.49	1.73 ± 0.50	−0.07	−0.28 to 0.14	0.51
QF ≤ 70%pred (*n*, %)	24 (27)	13 (32)	11 (23)			0.35
HGF (kg)	42 ± 14	43 ± 11	41 ± 16	2.0	−2.7 to 6.7	0.38
HGF ≤ 70%pred (*n*, %)	6 (6)	3 (7)	3 (6)			0.78
**Symptom experience**						
mMRC dyspnea score < 2 (*n* (%))	66 (84)	30 (83)	36 (84)			0.60
HADS Anxiety (score)	4 (1.0; 7.0)	3.0 (1.3; 7.0)	4.0 (1.0; 7.0)	−1.0	−1.4 to −0.6	0.88
HADS Depression (score)	2.0 (0.0; 4.3)	2.0 (0.0; 5.0)	2.0 (0.0; 4.0)	0.0	−0.2 to 0.2	0.92
SF-36						
- Physical component (score)	47 ± 10	1	47 ± 11	0	−3.9 to 3.9	0.93
- Mental component (score)	50 ± 9	1	51 ± 9	2	−1.8 to 5.8	0.40

Data are presented as mean ± SD median (Q1; Q3) or estimated marginal mean ± SE unless otherwise indicated. For physical activity outcomes, values for the total group (“All patients”) represent unadjusted means ± SD, while values for ICU and non-ICU groups are estimated marginal means adjusted for duration of daylight (ANCOVA). BMI: Body Mass Index; FEV_1_: Forced Expiratory Volume in 1 sec; DLCO: Diffusion Capacity; LOS: Length of Hospital Stay; NIVM: Non-Invasive Mechanical Ventilation; 6 MWD: Six-Minute Walk Distance; QF: Quadriceps Force; HGF: Handgrip Force; mMRC: Modified Medical Research Council; HADS: Hospital Anxiety Depression Score; SF-36: Short Form Health Survey; * adjusted for duration of daylight for PA outcomes.

## Data Availability

All data relevant to the study are included in the article.

## Supplementary Materials

The following supporting information can be downloaded at: https://www.mdpi.com/article/10.3390/jcm14176206/s1, Table S1: Bootstrapped ANCOVA results for primary PA outcomes for patients (*n* = 101) vs. healthy controls (*n* = 36).
