# Supplementary material for: Physical Activity and Physical Function One Year After Hospital Discharge for COVID-19
Source: J Clin Med. 2025 Sep 2;14(17):6206. doi: 10.3390/jcm14176206 (PMC12429844; doi:10.3390/jcm14176206)
Supplement: Supplementary file 1 [file jcm-14-06206-s001.zip › jcm-3754386-supplementary.pdf]

Supplementary Table 1. Bootstrapped ANCOVA results for primary PA outcomes for patients (n=101) vs healthy controls (n=36)

**Dependent variable**	**B**	**Standard Error**	**BCa 95% CI (Lower)**	**BCa 95% CI (Upper)**	**P-value**
Daily step count ($n.day^{-1}$)	1429	619	222	2521	**0.016**
Daily walking time ($min.day^{-1}$)	11.98	6.31	-1.43	25.04	0.074
Daily movement intensity ($m/s^2.day^{-1}$)	0.22	0.09	0.06	0.42	**0.014**

Data are presented as unstandardized regression coefficients (B) ± standard error, based on 1000 bootstrap samples. BCa = Bias-Corrected and Accelerated Confidence Interval. Analyses were adjusted for daylight duration.
